# Cases of high consequence infectious diseases identified in the UK, 1962–2023

**DOI:** 10.1099/jmm.0.001982

**Published:** 2025-03-12

**Authors:** Barry Atkinson, Mike Beadsworth, Jake Dunning

**Affiliations:** 1 Diagnostics and Pathogen Characterisation, UK Health Security Agency, Porton Down, Salisbury, UK; 2 NIHR Health Protection Research Unit in Emerging and Zoonotic Infections, UK; 3 Tropical and Infectious Disease Unit, Royal Liverpool University Hospital, Liverpool University Hospitals NHS Foundation Trust, Liverpool, UK; 4 Liverpool School of Tropical Medicine, Liverpool, UK; 5 Royal Free London NHS Foundation Trust, London, UK; 6 Pandemic Sciences Institute, University of Oxford, Oxford, UK

**Keywords:** avian influenza, Crimean-Congo haemorrhagic fever (CCHF), Ebola, high consequence infectious disease (HCID), human case, Lassa, Middle East respiratory syndrome (MERS), mpox, plague

## Abstract

*A corrigendum of this article has been published full details can be found at* https://doi.org/10.1099/jmm.0.002178

The management of patients with acute infectious diseases can present significant challenges, especially if the causative agent has a propensity for person-to-person transmission. In such cases, effective patient management is dependent on both rapid identification of disease and the provision of necessary medical care while adhering to suitable infection prevention and control measures to reduce the potential for onwards transmission. The UK has operated a defined system for managing patients with high consequence infectious diseases (HCIDs) since the 1970s, when protocols were first implemented following the first descriptions of several viral haemorrhagic fever diseases, including Marburg virus disease, Lassa fever and Ebola virus disease (EVD). While more than 200 people with HCIDs have been treated in UK hospitals since the 1970s, most of these patients had COVID-19 or mpox during the early phases of new public health emergencies of international concern (PHEICs), prior to their removal from the UK HCID list in March 2020 and June 2022, respectively. Excluding PHEICs, 26 patients have been treated in HCID treatment centres between 1962 and 2023: 10 patients with Lassa fever, 7 with mpox prior to the 2022 PHEIC, 4 with Middle East respiratory syndrome (MERS), 4 with EVD and 1 with Crimean-Congo haemorrhagic fever (CCHF). In total, 15 additional HCID patients were identified where treatment in a specialist centre did not occur due to retrospective diagnosis (4 patients with Lassa fever), mild or moderate illness [5 patients with avian influenza A(H5N1), 1 with MERS and 1 with CCHF] or death prior to transfer (2 patients with Lassa fever, 1 with CCHF and 1 with pneumonic plague). Here we summarize the UK HCID experience, including details about their detection, patient management and outcomes.

## Introduction

Providing effective treatment for patients with communicable diseases while simultaneously preventing onwards transmission is a central tenet of modern healthcare. While implementing suitable infection prevention and control (IPC) measures is paramount for all diseases with the ability to spread within healthcare systems and the wider community, certain diseases with higher case fatality rates, limited therapeutics available for their treatment and prevention and a propensity for person-to-person transmission require more stringent controls and response pathways.

The UK has utilized specialist isolation facilities to deal with high-risk communicable endemic diseases, including smallpox, for centuries; however, the introduction of routine vaccination programmes and implementation of modern IPC and hygiene measures resulted in a lower burden of disease and lower risk to the wider population from endemic pathogens. As the requirement for specialist regional centres to deal with endemic risks was reducing, several non-endemic pathogens were identified that could pose a risk to healthcare workers (HCWs) and the wider community if cases occurred in the UK. This risk was emphasized with the identification of the Marburg virus in 1967 when laboratory workers in three locations (two in Germany and one in the former Yugoslavia) developed a severe haemorrhagic fever following contact with African green monkeys (*Chlorocebus aethiops*) imported from Uganda. In total, 31 human cases of severe disease were reported (25 primary and 6 secondary), including 7 fatalities [1]. The severity of disease in affected cases caused concern for scientists, and both the US and the UK initiated programmes to build ‘maximum containment’ facilities to handle biological material that may pose significant risk to operators (the Biological Safety Level 4 suite at the Centers for Disease Control and Prevention in Atlanta and the Containment Level 4 suite at the UK Health Security Agency, Porton Down). While neither laboratory facility was operational during the response to the Marburg virus in Germany and the former Yugoslavia, both would be employed extensively in the response to the next emerging viral pathogen, Lassa virus. The initial discovery of the Lassa virus [2] not only altered our understanding of endemic infectious risks in West Africa, but the ramifications following two laboratory-acquired infections in the US had profound implications for modern biosafety, biocontainment and clinical management of high consequence infectious diseases (HCIDs) [3,4]. This impact was particularly notable in the UK, as several cases of Lassa fever were identified subsequently in travellers; these identifications would lay a foundation for the formalization of HCID clinical care, not only for cases of Lassa fever but also for other HCIDs that would subsequently be identified and/or classified.

The management of patients in the UK with non-endemic diseases that pose significant risk to both HCWs and the general public was primarily undertaken at Coppetts Wood Hospital in London, in conjunction with infectious disease specialists from the Royal Free Hospital, London. Coppetts Wood was one of the UK’s largest specialist isolation centres and was used extensively in the 19th and 20th centuries to provide care to patients with infectious diseases endemic at the time, such as smallpox, tuberculosis and diphtheria. As the burden from these endemic diseases decreased in the 20th century, Coppetts Wood became the logical choice for the management of newly discovered high consequence pathogens. The specialist HCID services offered by the Coppetts Wood High Security Infectious Diseases Unit were eventually transferred to a new high-level isolation unit within the Royal Free Hospital in 2008 [5,6]. While Coppetts Wood was the principal site for treating HCID patients until 2008, other specialist isolation units were utilized on occasion when logistical factors superseded preference, especially in relation to patient transport.

**Fig. 1.**
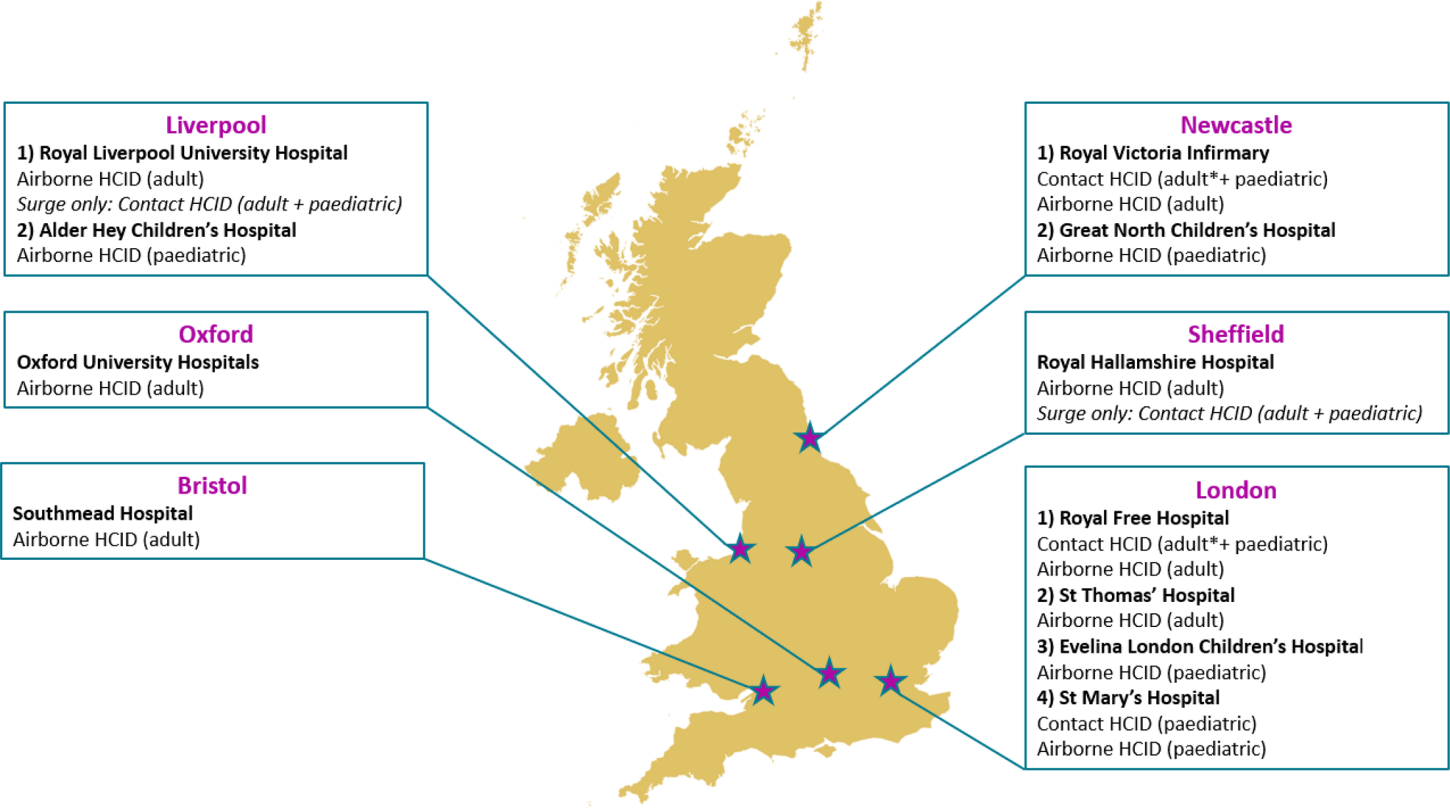
Locations of HCID Treatment Centres in the UK. Treatment is provided using a ward-based system by HCWs wearing defined PPE unless otherwise indicated (^*^=treatment provided using Trexler patient isolator). Italics indicate the hospital used to provide surge capacity in an outbreak scenario that exceeds established capacity.

Between 2016 and 2018, NHS England and Public Health England (now UK Health Security Agency) ran a joint development programme to strengthen the end-to-end management of patients with suspected and confirmed high-risk infectious diseases. This was in response to recent public health emergencies of international concern (PHEICs), including the 2013–2016 West Africa Ebola virus disease (EVD) epidemic and cases and outbreaks of Middle East respiratory syndrome (MERS). The programme introduced and defined the term HCID in the UK and resulted in national commissioning of additional specialist infectious disease centres in England to provide specialist, safe care to adult and paediatric patients with HCIDs. The programme identified 15 distinct HCIDs and assigned them as either ‘contact HCID’ or ‘airborne’ HCID [7] (Table 1). The distinction between ‘contact’ and ‘airborne’ is related to the engineering and IPC measures required for HCWs to safely interact and manage patients, according to how the diseases are transmitted between humans in the community [8]. As shown in Fig. 1, there are 11 treatment centres located in 6 cities across England capable of receiving patients with HCIDs. These centres offer logistical flexibility along with surge capacity should multiple cases be identified. Cases identified in Scotland, Wales and Northern Ireland may also be transferred to the centres in England in the absence of suitable local HCID facilities.

**Table 1.** List of HCIDs currently recognized in the UK (accurate as of 31 December 2023)

Contact HCIDs [Causative agent]	Airborne HCIDs [Causative agent]
Argentinian haemorrhagic fever [Junin virus]	Andes virus infection [Andes virus]
Bolivian haemorrhagic fever [Machupo virus]	Avian influenza* [Influenza A H5N1, H5N6, H7N7, H7N9 viruses]
Crimean-Congo haemorrhagic fever (CCHF) [CCHF virus]	MERS [MERS coronavirus]
EVD [Ebola virus]	Mpox [Monkeypox virus – Clade I only†]
Lassa fever [Lassa virus]	Nipah virus infection [Nipah virus]
Lujo virus disease [Lujo virus]	Pneumonic plague [*Yersinia pestis*^‡^]
Marburg virus disease [Marburg virus]	Severe acute respiratory syndrome (SARS) [SARS coronavirus]
Severe fever with thrombocytopaenia (SFTS) [SFTS virus; aka Dabie Mountain virus]	
**Diseases formerly listed as HCIDs [year delisted]**	
COVID-19 [2020]	
Mpox – Clade IIb [2022]	
Mpox – Clade IIa [2023]	

* Only avian influenza resulting from infection with H5N1, H5N6, H7N7 or H7N9 influenza A viruses are considered as HCIDs.

† Mpox resulting from infection with clade IIa and IIb monkeypox viruses are no longer listed as HCIDs.

‡ Only the pneumonic presentation of plague is classified as an HCID.

**Fig. 2.**
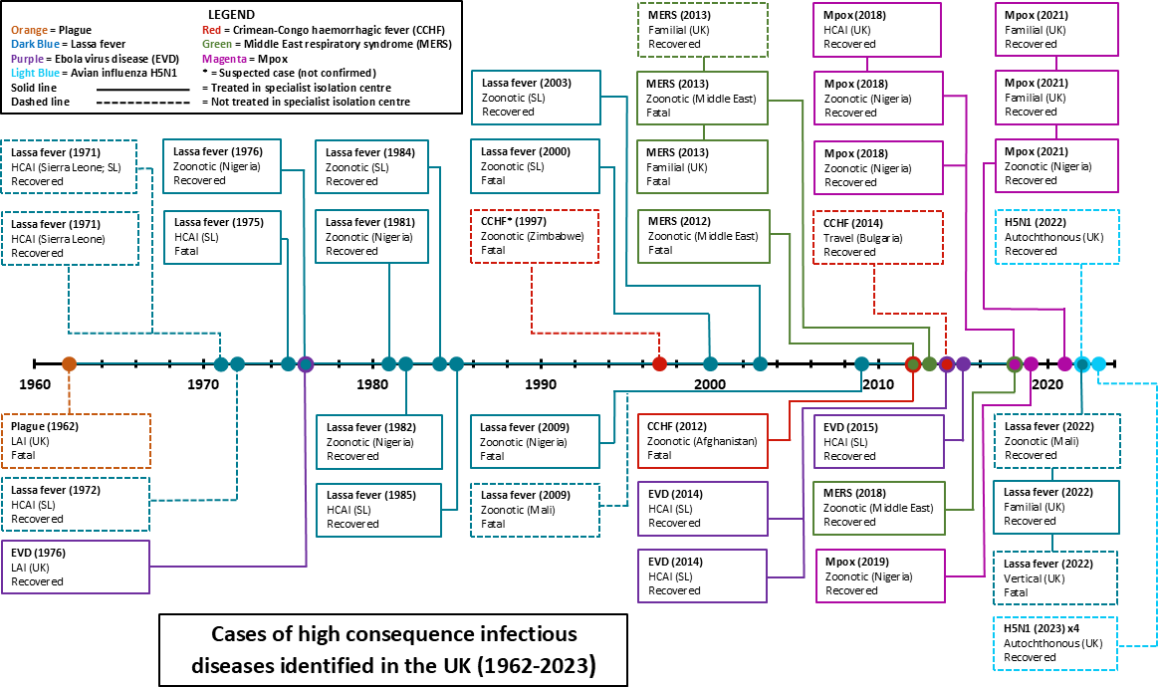
Timeline of HCID cases identified in the UK (1962–2023). HCAI=healthcare acquired infection; LAI=laboratory acquired infection. Cases labelled as ‘zoonotic’ indicate infection likely occurred from interaction with infected animal or vector in an endemic region; however, the exact mode of acquisition is not typically known.

Since 1975, more than 200 patients with HCIDs have been managed at specialist centres in the UK; however, the majority of patients had either COVID-19 or mpox caused by clade IIb monkeypox virus, and these diseases are no longer considered HCIDs in the UK. For the other diseases that remain on the HCID list on 1 January 2024, 26 patients have been treated in a UK HCID Treatment Centre, in addition to 15 patients with HCIDs who did not require transfer to an HCID Treatment Centre (Fig. 2). Reasons for not transferring a patient to an HCID Treatment Centre include retrospective diagnosis in a recovered and/or non-infectious patient; moderate, mild or asymptomatic disease that can be managed and monitored safely elsewhere; death having occurred before transfer could take place.

The identification, assessment and safe management of patients suspected to have HCIDs is the responsibility of all National Health Service (NHS) hospitals with emergency departments and/or the ability to admit acutely unwell patients. A balance needs to be struck between ensuring detection of HCIDs, which generally are rare diseases in the UK, without delaying the identification and management of more common, and sometimes serious, travel-associated infections and other acute illnesses which can present with similar symptoms [9,12]. This can be challenging for busy acute services, but online tools are available to assist clinicians [7], as well as advice and support from infection specialists and the UK Imported Fever Service (IFS) [13]. The management of suspected HCID cases has been discussed elsewhere [8]. This report details reported cases of HCIDs reported in the UK, including their detection, summarized clinical management and outcomes.

## Pneumonic plague: 1 case, 1 death

Plague caused by *Yersinia pestis* infection, specifically the pneumonic form of the disease, is an outlier on the current HCID list; not only is it the sole bacterial disease, but it is also the only disease that was once endemic in the UK. The last known natural autochthonous case of plague in the UK occurred in 1918 [14], but a case occurred subsequently in 1962, when a scientist developed pneumonic plague through a laboratory-acquired infection [15]. The patient was not aware of the laboratory exposure, meaning initial medical treatment was for a non-specific viral infection with no specialist IPC measures implemented; they died in a local hospital on the day that a diagnosis of pneumonic plague was made based on clinical symptoms and a suspected laboratory exposure.

## Lassa fever: 16 cases, 5 deaths

The first three cases of Lassa fever diagnosed in the UK were all non-fatal cases in HCWs who had recently returned to the UK from Sierra Leone. Two of the cases were diagnosed in 1971 [16], and the third in 1972 [17]. None of these cases were treated in a treatment centre, as all three were diagnosed retrospectively during scientific investigations into the potential impact of the newly identified disease.

Six cases of Lassa fever received care in hospitals between 1975 and 1985: a physician working in Sierra Leone who died 48 h after repatriation to the UK (Hospital for Tropical Diseases, London, 1975; fatal) [18,19], an engineer working in Nigeria (Coppetts Wood Hospital, 1976; survived) [20], a Nigerian teacher visiting the UK (Coppetts Wood Hospital, 1981–1982; survived) [21,22], a Nigerian resident visiting the UK (Coppetts Wood Hospital, 1982; survived) [23], a geologist working in Sierra Leone (Coppetts Wood Hospital, 1984; survived) [24] and a midwife working in Nigeria (Ham Green Hospital, Bristol, 1985; survived) [25]. Experience gained from managing these six cases helped inform the safe management of future HCID cases in the UK, including new advancements in IPC measures and patient transportation, such as the use of patient isolators and military aircraft to safely move eligible patients to the UK to receive care when necessary [26].

Fifteen years passed before another case of Lassa fever was registered in the UK, in a British aid worker who acquired Lassa fever while working in Sierra Leone (Coppetts Wood Hospital, 2000; fatal) [27]. Three more cases were reported in the 2000s: a British soldier serving in Sierra Leone (Coppetts Wood Hospital, 2003; survived) [28], a British resident returning from a visit to Nigeria (Royal Free Hospital, 2009; fatal) [29] and a British resident medically evacuated from Mali with falciparum malaria not responding to treatment (2009; fatal – died prior to planned transfer to Royal Free Hospital) [30].

The most recent cases of Lassa fever detected in the UK were diagnosed in 2022, affecting three individuals from the same family. The index case acquired the Lassa virus whilst visiting his native Mali, becoming unwell on return to the UK. They were admitted to hospital with unexplained fever, received empirical antibiotics and recovered after a few days. Lassa fever was not considered at the time of illness, and they did not undergo diagnostic testing for Lassa fever while in hospital. The index case’s partner, who was pregnant, also contracted Lassa fever despite not travelling to Mali, inferring autochthonous transmission in the UK. The female patient became unwell during her third trimester of pregnancy and developed critical illness after her baby was delivered in hospital. The baby then became critically ill days later, at which point the father’s travel history and recent febrile illness were recognized. qPCR testing for viral haemorrhagic fever viruses was performed on blood samples from the father, the mother and the baby, and all samples tested positive for Lassa virus. The mother was transferred to the Royal Free Hospital for ongoing critical care and survived. The baby was moribund at the time of the Lassa fever diagnosis; therefore, rather than being transferred to an HCID Treatment Centre, the baby received end-of-life care with appropriate enhanced IPC precautions implemented at the hospital where they were born (manuscript in preparation).

## EVD: 4 cases, 0 deaths

The first UK case of EVD occurred in 1976 following a laboratory-acquired infection caused by a percutaneous sharps (‘needlestick’) injury during investigations into the recently identified Ebola Sudan virus. Immediate investigations did not find any evidence of skin penetration; however, after developing non-specific febrile symptoms, the patient was transferred to Coppetts Wood Hospital for monitoring. The patient subsequently developed a short but moderately severe course of EVD. They were discharged after receiving care for 2 weeks, but full recovery took 3 months. This case represents the first use of a Trexler patient isolator for managing patients with a contact HCID [31].

The other three cases of EVD reported in the UK were all associated with the West Africa Ebola epidemic of 2013–2016. All three patients were HCWs who had been supporting the international outbreak response in Sierra Leone. Two were medically evacuated to the UK for treatment at the Royal Free Hospital, while the third developed symptoms upon return to the UK and was transferred to the Royal Free Hospital following diagnosis [32]. While all three patients survived, one patient suffered notable sequelae and episodes of recrudescence, including Ebolavirus meningoencephalitis 9 months after their initial EVD illness, which necessitated aeromedical transfer from a hospital in Scotland and readmission to the Royal Free Hospital [33]. This case was the first documented report of late severe relapse of the Ebola virus with viral RNA re-detectable in blood and one of only a few reported cases of relapse involving neurological disease and virus detectable in cerebrospinal fluid.

## Crimean-Congo haemorrhagic fever: 3 cases, 2 deaths

The first confirmed case of Crimean-Congo haemorrhagic fever (CCHF) in the UK occurred in 2012 when a traveller from Afghanistan presented at an emergency department in Glasgow with fever and several haemorrhagic manifestations, including haematemesis, bloody diarrhoea and conjunctival suffusion [34]. Despite reporting no contact with ticks or animals, CCHF was suspected and confirmed by qPCR shortly after sample collection [35]. The patient was transferred to the Royal Free Hospital following diagnosis; however, despite rapid identification of the causative agent, there was further clinical deterioration and the patient died 4 days after initial presentation [34].

A second confirmed case occurred less than 2 years later in a traveller returning from a holiday to Bulgaria. The clinical presentation observed in this case was less severe than for the 2012 case, and the patient was recovering by the time they were diagnosed after presentation to a UK hospital [36]. A decision was made to continue treatment in the isolation room at the local hospital, with appropriate IPC measures in place, rather than transferring the recovering patient to the Royal Free Hospital. This decision considered the lack of overt haemorrhagic manifestations, the overall clinical improvement and the ability to implement enhanced IPC measures *in situ*.

In 2022, a case of CCHF was initially reported in the UK in a woman with a history of recent travel to Central Asia. The patient was transferred to the Royal Free Hospital for further care [37]; however, the CCHF diagnosis was not confirmed on testing of subsequent patient samples, and an alternative diagnosis was made on further investigations. The patient has therefore been removed from official statistics [38]. Accordingly, this case is not included as a CCHF case in this report.

While only two cases of CCHF have been officially reported in the UK up to 2023, one additional ‘suspected’ case requires consideration. This suspected case occurred in late 1997 when a UK traveller developed a serious illness suspected to be either malaria or typhoid fever upon return from Zimbabwe. They were treated in a local hospital, but their condition deteriorated rapidly, including the development of thrombocytopaenia, melaena and haemorrhagic rash, prompting suspicion of CCHF. Samples collected shortly prior to death were strongly positive for CCHF-specific IgG and weakly positive for IgM; however, viral isolation attempts were negative [39]. While these analyses are suggestive of CCHF, the lack of paired sera to assess an increase in antibody titre and the absence of confirmatory tests resulted in this being reported as a suspected rather than confirmed case. This suspected case has been counted in statistics for this analysis.

## MERS: 5 cases, 3 deaths

The first case of MERS identified in the UK occurred in September 2012 in a Qatari patient with respiratory distress and a recent travel history to Saudi Arabia. They were medically evacuated from Qatar to the UK and admitted to a specialist intensive care facility in London, at which point a diagnosis of MERS was made. At the time, this was the second case of MERS reported globally shortly after the first case in Saudi Arabia in the same month [40]. Unfortunately, the patient died despite receiving 231 days of extracorporeal membrane oxygenation (ECMO) treatment for severe acute respiratory failure and late complications of MERS pneumonitis [41].

Three additional cases of MERS were identified in 2013 within members of the same extended family. The index case of this family cluster had recent travel to Pakistan and Saudi Arabia, and they became ill shortly before returning to the UK. Following arrival in the UK, their symptoms worsened, and they were admitted to a local hospital specializing in treatment of respiratory diseases. The patient received invasive mechanical ventilation for management of severe acute respiratory failure and subsequently received ECMO [42]. They were diagnosed with MERS but ultimately succumbed to this infection despite significant medical interventions [43]. Following the diagnosis, contact tracing identified two further cases in family members who had not travelled abroad. The first was a family member living in the same household as the index case; the second was a member of the extended family living in a separate household but with close contact with the index case. The patient in the extended family had mild illness and was managed without admission to a specialist facility. By contrast, the family member within the same household developed severe MERS, possibly exacerbated by an underlying health condition requiring treatment that likely resulted in immunosuppression. This patient required hospitalization and critical care, including ECMO, but they died 8 days after admission to hospital [42].

The fifth case of MERS identified in the UK occurred in 2018 when a traveller from the Middle East was diagnosed while visiting the UK. They were initially admitted to a local hospital but, following diagnosis of MERS, they were transferred to, and managed at, the Royal Liverpool University Hospital, a newly commissioned Airborne HCID centre [44]. The patient recovered from their illness and no subsequent cases were identified in close contacts [43].

## Mpox (pre-PHEIC): 7 cases, 0 deaths

Seven cases of clade IIb mpox (formerly known as monkeypox) were identified prior to the PHEIC beginning in May 2022. Four cases had recent travel to Nigeria, where cases of mpox had recently been reported for the first time since the 1970s [45,46]; three additional cases resulted following person-to-person transmission in the UK.

The first two UK mpox cases were identified a few days apart in September 2018 despite these cases being epidemiologically unconnected [47,48]. Both cases had recent Nigerian travel histories (Patient 1 was a Nigerian naval officer visiting the UK for training, and Patient 2 was a British resident returning from a trip to Nigeria). Both patients were transferred to Airborne HCID Treatment Centres following diagnosis (Patient 1 to the Royal Free Hospital, London, and Patient 2 to the Royal Liverpool University Hospital). Both patients fully recovered. The third case identified resulted following a healthcare-associated infection of an HCW who changed the bed linen from Patient 2 prior to diagnosis and implementation of mpox-appropriate IPC measures [49]. This patient (Patient 3) was admitted to the Royal Victoria Infirmary in Newcastle; they also made a full recovery.

A further case (Patient 4) was identified in 2019 following travel to Nigeria [50]. Upon diagnosis, the patient was transferred to the Airborne HCID Treatment Centre at St Thomas’ Hospital, London. The patient had a brief clinical and virological relapse 6 weeks after being discharged but eventually recovered fully.

The final three cases (Patients 5–7) occurred in a family cluster identified in 2021 following travel by the index case to Nigeria. Case 5 developed symptoms 2 days after returning to the UK, with symptom onset occurring 19 days later for Case 6 and 33 days later for Patient 7 [51]. All were treated at the Royal Liverpool University Hospital, with support from Alder Hey Children’s Hospital, and all made a full recovery.

All seven cases resulted from infection with Clade IIb monkeypox virus, one of four major clades of monkeypox virus currently identified along with Clade Ia, Clade Ib and Clade IIa. Following the mpox PHEIC declared in 2022, the UK’s Airborne HCID list was amended, with only Clade I mpox (Ia and Ib) remaining as an HCID, meaning future Clade IIa and IIb mpox cases identified in England will no longer be managed solely by the Airborne HCID Network. The rationale for declassification of both Clade IIa and IIb mpox is discussed further in the ‘Cases managed during PHEICs’ section.

## Avian Influenza (H5N1): 5 cases, 0 deaths

The first confirmed human case of avian influenza occurred in 2022 following detection of the highly pathogenic avian influenza H5N1 virus in ducks kept at a domestic property. In addition to numerous positive ducks in this flock, the property owner also tested positive for the avian influenza H5N1 virus but remained asymptomatic throughout their infection and, as they could safely isolate at home while receiving regular clinical monitoring, did not require transfer to a specialist treatment centre [52]. Asymptomatic surveillance studies targeting at-risk individuals linked to poultry outbreaks identified four positive cases in 2023; however, the low levels of nucleic acid identified combined with epidemiological factors raise the possibility of environmental contamination in the upper respiratory tract rather than human infection [53].

## Cases managed during PHEICs

### Severe acute respiratory syndrome: 4 cases, 0 deaths

In 2003, prior to a case of severe acute respiratory syndrome (SARS) being identified in the UK, bespoke guidance was issued to manage both potential and confirmed cases of SARS and to mitigate the potential for overwhelming specialist treatment centres should sustained transmission occur in the UK. This guidance recommended that mild cases of illness should be treated at home with severely ill cases managed in an isolated room within a suitable local hospital with defined IPC measures applied [54,55]. More than 400 possible case reports were recorded in the UK within a 5 month period in 2003, of which 8 were reported to the World Health Organization as probable cases; 4 of these 8 cases were later declassified following confirmational analysis [56]. There were no fatal outcomes recorded in the UK.

Although no cases from the 2003 outbreak were treated within treatment centres, and despite no reports of SARS cases internationally since 2004 [57], SARS is included on the UK’s Airborne HCID list because cases remain reportable under the International Health Regulations (2005); therefore, if future SARS outbreaks occur, hospitalized patients in England would be managed in Airborne HCID Treatment Centres.

### COVID-19: unknown case count

COVID-19 was added to the Airborne HCID list in early 2020 as an ‘interim recommendation’. Accordingly, initial cases in England – regardless of severities of illness – were managed in Airborne HCID Treatment Centres. This provided an opportunity to provide optimal IPC measures, with the aim of trying to reduce onwards transmission; however, the propensity for mild and asymptomatic case presentations, plus a gradual increase in more severe cases, resulted in the UK case count quickly overwhelming the finite capacity of the network of Airborne HCID Treatment Centres.

As case numbers became unmanageable within the HCID network, both suspected and confirmed cases started to be managed elsewhere. Cases were triaged initially, with patients with severe disease or with underlying comorbidities being admitted or transferred to the Airborne HCID Treatment Centres, but it soon became clear that even this approach would be unsustainable in a pandemic.

In anticipation of a worsening UK outbreak and a possible pandemic declaration, the UK government announced its national Coronavirus Action Plan on 3 March 2020, which included case management by the entire NHS [58]. COVID-19 was removed from the Airborne HCID list on 19 March 2020, following a review of accumulated global epidemiological data measured against the HCID framework criteria [7]. Due to the stress on the healthcare system at this point, including a 3 month national lockdown that would begin days after declassification, it is not known exactly how many patients were admitted to HCID treatment centres or what their outcomes were.

### Mpox: unknown case count

More than 3000 cases of Clade IIb mpox were identified in the UK during the 2022–2023 PHEIC. Initial cases were treated in Airborne HCID Treatment Centres, regardless of severity of illness, as mpox caused by any virus clade was an Airborne HCID at the start of this outbreak. As the number of cases in England escalated, particularly in London, it became apparent that the capacity of the network of Airborne HCID treatment centres would be exceeded, even with expansion of internal bed capacities and additional support from other infectious diseases centres. Therefore, approaches to patient management changed over time, with mild cases capable of self-isolating remaining at home (with ‘virtual’ clinical and public health monitoring where necessary), cases unable to isolate safely being admitted to non-HCID centres (other specialist infectious disease hospitals) and the more severe cases being managed by the Airborne HCID Treatment Centres.

Following a review of accumulated clinical and epidemiological data from UK and international cases diagnosed in May and June 2022, Clade IIb mpox was removed from the Airborne HCID list in July 2022 [59]. An accurate case count for the number of patients treated in Airborne HCID Centres is not available, in part because additional surge mechanisms were implemented to manage patients requiring hospitalization. While the majority of UK cases were uncomplicated, cases of severe disease were identified, including a case of encephalitis with transverse myelitis [60]. However, there were no reported deaths within the UK, and the Airborne HCID network continued to provide oversight throughout the outbreak.

In January 2023, Clade IIa mpox was also removed from the HCID list [61]. Although no Clade IIa cases have been detected in the UK, a Clade IIa outbreak relating to imported rodents was identified in the US in 2003. Of the 71 cases identified, 2 presented with serious clinical illness (both children) and the majority of hospitalizations were as a precautionary measure [62].

As of 2024, only Clade I mpox is considered an HCID in the UK [63].

## Smallpox

Smallpox is not listed as an HCID, as this disease was declared eradicated by the World Health Assembly in 1980. However, there is a non-zero risk of human cases being detected in the future as infectious viral material is maintained in two specialist facilities in addition to the potential for rescuing infectious virus through synthetic biology. In 2017, infectious horsepox virus was rescued from genetic sequences through large-scale gene synthesis [64], demonstrating the potential for variola virus to be synthesized and used for nefarious purposes. If a case of smallpox were to be detected in the UK, the patient(s) would most likely be managed within the HCID network and possibly within a Trexler isolator to achieve the highest level of biocontainment.

Smallpox ceased to be considered an endemic disease in the UK in the 1930s; however, sporadic outbreaks occurred until the 1960s, typically linked to imported cases [65,66], with cases managed in designated isolation hospitals. Although the last endemic case of smallpox reported globally occurred in Somalia in 1977, two further cases occurred in the UK after this date. The index case, Janet Parker, was a photographer working in a university medical school that conducted smallpox research, and the second case was her mother. Janet Parker died on 11 September 1978 and became the last person known to have died from smallpox; her mother survived her illness and is the last known person to contract smallpox. The official report into this outbreak concluded that Janet Parker was likely infected through a virus that was handled at the medical school, either via the ventilation system or by fomite transfer [67].

As smallpox is not currently a listed HCID, these cases are not reflected in this analysis.

## HCIDs not yet seen in the UK

Up to the end of 2023, only 7 of the 15 listed HCID diseases have been detected in cases within the UK [Lassa fever, EVD, CCHF, avian influenza A(H5N1), MERS, SARS and pneumonic plague]. The eight HCIDs that have not yet been diagnosed in the UK are Argentinian haemorrhagic fever (caused by Junin virus infection), Bolivian haemorrhagic fever (caused by Machupo virus infection), Lujo virus disease, Marburg virus disease, Andes hantavirus infection, Nipah virus infection, mpox caused by clade I virus and severe fever with thrombocytopaenia syndrome (SFTS, caused by SFTS virus; also known as Dabie Mountain virus). In addition, while the UK has detected avian influenza A(H5N1) in five people (all lineage 2.3.4.4b), human infections with avian influenza viruses A(H5N6), A(H7N7) and A(H7N9) have not been detected in the UK. Despite the absence of some HCID cases in the UK, imported cases have been reported elsewhere, including Argentinian haemorrhagic fever (Belgium ex Argentina [68]), Marburg virus disease (Netherlands ex Uganda [69] and USA ex Uganda [70]), Lujo virus disease (South Africa ex Zambia [71]) and Andes hantavirus infection (Switzerland ex Chile [72] and USA ex Argentina/Chile [73]).

## Discussion

Although none of the currently listed HCIDs is considered endemic in the UK, the potential for HCID cases to be identified in the UK remains ever present due to multiple factors, including the increasing frequency of global travel and people returning to the UK after assisting with humanitarian, scientific and medical response efforts for HCID outbreaks in other countries. Rapid diagnosis and effective clinical management of HCID cases are of paramount importance, not only to improve the chances of a positive patient outcome but also to limit the potential for onwards transmission within healthcare systems and the wider community. Despite the absence of HCID pathogens in the UK, 41 HCID cases have been reported between 1967 and 2023, including 26 that received specialist care in HCID Treatment Centres. This figure does not include hundreds of patients who received care in Airborne HCID Treatment Centres during the COVID-19 and mpox PHEICs during periods when these diseases were classified as HCIDs.

Interestingly, 14/41 (34.1%) of HCID cases identified were contracted in the UK despite the absence of endemic HCID pathogens. These 14 cases include 5 cases of familial transmission (2 each for mpox and MERS and 1 for Lassa fever), 2 laboratory-acquired infections (pneumonic plague and EVD), 1 healthcare-acquired infection (mpox), 1 case of vertical transmission (Lassa fever) and 5 cases of autochthonous transmission (H5N1 avian influenza, which may be present in wild birds in the UK but is not considered endemic [74]). This highlights the importance of not assuming all potential HCID cases require a relevant overseas travel history, although 6 of these 14 cases were contacts of cases who had acquired their infections in countries with known risks of the respective HCIDs.

The family cluster of Lassa fever cases in 2022, where only the index case had travelled and their infection was not diagnosed initially, demonstrates the need to consider exposure to undiagnosed cases that may have recovered by the time their contacts develop symptoms. This experience suggests that, for a patient with severe acute febrile illness that lacks a diagnosis despite testing for common pathogens, enquiring about recent illnesses and travel histories of their close contacts has the potential to reveal a previously unrecognized and unexpected exposure to an HCID pathogen, triggering the necessary diagnostic testing that might not otherwise be requested.

Provision of healthcare facilities that provide specialist care to patients while preventing transmission through enhanced IPC requires specific financial and human resources, along with appropriate physical infrastructure. While it is not possible to know what would have happened if the cases described here had all been treated in standard hospitals, there is evidence of what can happen when HCID incidents are not managed optimally. For example, a nosocomial outbreak of CCHF occurred in Afghanistan in 2023, where healthcare treatment of a single CCHF-positive patient resulted in an additional 48 cases, including 13 HCWs and 1 patient being treated for an unrelated issue [75]. Similarly, one travel-associated case of MERS identified in South Korea resulted in a significant outbreak lasting 2 months, with 186 confirmed cases and 28 fatalities [76]. The estimated financial loss to the country’s travel and tourism industry from that 2015 outbreak was 2.6 billion US dollars [77]. Suboptimal clinical pathways and IPC measures also resulted in three HCWs becoming infected with EVD in the USA and Europe [32].

While the risk for onwards transmission in hospitals and the wider community is established for many HCID pathogens, the risk for rarer HCIDs remains more hypothetical due to a scarcity of primary data. For this reason, a reasonable worst-case scenario is considered for mitigation purposes until substantial primary evidence is available. In addition, many of the case reports that help inform UK planning for HCIDs are from lower- or middle-income countries where diagnostics, contact tracing and patient management resources may differ from those in the UK; therefore, frequent review of guidelines and control measures is required, which may result in new diseases being added to the UK HCID lists or in HCIDs being delisted as our understanding of human disease develops. Importantly, the HCID system in England specifically excludes pandemics caused by respiratory viruses, which have their own dedicated response plans and, necessarily, much larger systems of clinical care.

Real-time monitoring of newly emerged data on HCIDs has not only led to the addition and removal of certain diseases from the HCID list but also identified a requirement for HCID treatment centres that specialize in managing patients with airborne HCIDs. England’s network of commissioned Airborne HCID Treatment Centres, established in 2018, has only managed one case of MERS (as of the end of 2023), but the network has managed many more cases of clade IIb mpox and COVID-19 when these diseases were classified as HCIDs. Such specialist management in the early phases of the respective PHEICs brought multiple benefits, including helping attempts to delay spread and/or prevent additional cases (a stated strategic aim in the public health response to these diseases), providing access to specific treatments and learning more about emerging infections and how to manage them through active clinical research. Additionally, the surge responses required for managing COVID-19 cases in early 2020, and clade IIb mpox in 2022, have provided the HCID treatment centres with valuable practical experience and identified the need for ongoing, adaptable, emergency preparedness, resilience and response systems for HCIDs in the NHS in the UK.

It is reasonable to expect that the NHS will need to provide care to more patients with HCIDs in the future and that the number of HCID outbreak cases may increase over time, particularly with the growth and movement of human populations, changes in the animal–human interface and the predicted wide-ranging effects of climate change. This may include as yet unidentified infectious diseases which could be added to HCID lists in the future, such as a ‘disease X’. What is harder to predict is the timing, size and impact of future HCID outbreaks affecting the UK, even for the HCIDs currently listed. There may also be an alteration in the frequency of detection for specific HCIDs; while only three cases of CCHF were detected in UK travellers in this review period, two European countries reported their first autochthonous cases of CCHF in recent years (Spain in 2013 [78] with numerous human cases reported since and Portugal in 2024 [79]). In addition, CCHF virus-positive ticks were identified for the first time in France in 2022 and 2023 [80]. This changing epidemiology in relation to areas of endemicity subsequently affects the likelihood of imported cases due to increased areas of risk and, in this instance, for regions that are popular holiday destinations for UK travellers.

In addition to online guidance and risk assessments for assessing the likelihood of HCIDs in individual patients [7], the UK has an IFS that offers 24/7 clinical, diagnostic and infection control advice to medical professionals managing recent travellers with fever [13], including those who may have an HCID. The IFS is also the pathway for arranging testing of HCIDs through the Rare and Imported Pathogens Laboratory at UKHSA Porton Down, with the exception of respiratory HCIDs, which are tested at the Virus Research Department at UKHSA Colindale. Although HCIDs are rare diseases, these diagnostic services and tools, in combination with the NHS England HCID Networks, offer the ability to detect cases rapidly and provide optimal patient care and IPC, with the aim of achieving the best clinical outcomes for the affected individuals while minimizing the risk of transmission to others and avoiding disruption of routine healthcare services. Based on the UK’s experience of HCID cases from the last 60 years, as described here, there is value in maintaining and strengthening public health and clinical systems for managing HCIDs, albeit in a proportionate way.

## Abbreviations

CCHF: Crimean-Congo haemorrhagic fever
ECMO: extracorporeal membrane oxygenation
EVD: Ebola virus disease
HCIDs: high consequence infectious diseases
HCWs: healthcare workers
IFS: Imported Fever Service
IPC: infection prevention and control
MERS: Middle East respiratory syndrome
NHS: National Health Service
PHEICs: public health emergencies of international concern
SARS: severe acute respiratory syndrome
SFTS: severe fever with thrombocytopaenia syndrome
